# A functional variant of Fcγ receptor IIIA is associated with rheumatoid arthritis in individuals who are positive for anti-glucose-6-phosphate isomerase antibodies

**DOI:** 10.1186/ar1802

**Published:** 2005-08-11

**Authors:** Isao Matsumoto, Hua Zhang, Yoshifumi Muraki, Taichi Hayashi, Takanori Yasukochi, Yuko Kori, Daisuke Goto, Satoshi Ito, Akito Tsutsumi, Takayuki Sumida

**Affiliations:** 1 Clinical Immunology, University of Tsukuba, University of Tsukuba, Ibaraki, Japan; 2 PRESTO, Japan Science and Technology Agency, Saitama, Japan

## Abstract

Anti-glucose-6-phosphate isomerase (GPI) antibodies are known to be arthritogenic autoantibodies in K/B×N mice, although some groups have reported that few healthy humans retain these antibodies. The expression of Fcγ receptors (FcγRs) is genetically regulated and has strong implications for the development of experimental arthritis. The interaction between immune complexes and FcγRs might therefore be involved in the pathogenesis of some arthritic conditions. To explore the relationship between functional polymorphisms in FcγRs (*FCGR3A*-158V/F and *FCGR2A*-131H/R) and arthritis in individuals positive for anti-GPI antibodies, we evaluated these individuals with respect to *FCGR *genotype. Genotyping for *FCGR3A*-158V/F and *FCGR2A*-131H/R was performed by PCR amplification of the polymorphic site, followed by site specific restriction digestion using the genome of 187 Japanese patients with rheumatoid arthritis (including 23 who were anti-GPI antibody positive) and 158 Japanese healthy individuals (including nine who were anti-GPI antibody positive). We report here on the association of *FCGR3A*-158V/F functional polymorphism with anti-GPI antibody positive status. Eight out of nine healthy individuals who were positive for anti-GPI antibodies possessed the homozygous, low affinity genotype *FCGR3A*-158F (odds ratio = 0.09, 95% confidence interval 0.01–0.89; *P *= 0.0199), and probably were 'protected' from arthritogenic antibodies. Moreover, among those who were homozygous for the high affinity genotype FCGR3A-158V/V, there were clear differences in anti-human and anti-rabbit GPI titres between patients with rheumatoid arthritis and healthy subjects (*P *= 0.0027 and *P *= 0.0015, respectively). Our findings provide a molecular model of the genetic regulation of autoantibody-induced arthritis by allele-specific affinity of the FcγRs.

## Introduction

Rheumatoid arthritis (RA) is a heterogeneous autoimmune disease that is characterized by chronic inflammatory polyarthritis [1]. One of the characteristic features of RA is the expression of several autoantibodies. The presence of such autoantibodies (e.g. rheumatoid factor [RF]), identified by screening, is commonly used as a diagnostic marker, although the pathogenic role played by autoantibodies in RA remains a mystery.

Fcγ receptors (FcγRs) play a pivotal role in the reaction between immune complex and myeloid cells. Three FcγR types have been identified in mice and humans (FcγRI, FcγRII and FcγRIII). In mouse arthritis models, FcγRIII deficient hosts exhibit resistance to collagen type II induced arthritis and anti-glucose-6-phosphate isomerase (GPI) antibody induced arthritis [2,3], suggesting that FcγRIII is indispensible in autoantibody dependent arthritis. In humans FcγRs are encoded by eight genes, and the genes encoding the low affinity FcγRs (*FCGR2A*, *FCGR3A, FCGR2C*, *FCGR3B *and *FCGR2B*) are located within a gene cluster on chromosome 1q22-23. Of these FcγRs, FcγRIIIa and FcγRIIa are known to be stimulatory receptors. Various genetic polymorphisms of these receptors were reported to be associated with several autoimmune diseases [4,5], one of which is a polymorphism in *FCGR3A*, with either a phenylalanine (F) or a valine (V) at amino acid position 158 [6,7]. Moreover, based on findings from a co-crystalization study with IgG_1 _and FcγRIIIa [8], this residue directly interacts with the lower hinge region of IgG_1_, suggesting strong binding between IgG_1 _and FcγRIIIa-158V on both natural killer cells and macrophages. For *FCGR2A *genes, a polymorphism at position 131 (with either histidine [H] or arginine [R]) alters the ability of the receptor to bind to certain IgG subclasses [9,10].

In RA patients, *FCG3A*-158V/F polymorphisms were reported to be frequent in UK Caucasian, North Indian and Pakistani individuals [11,12], but not in Japanese, Spanish and French individuals [13-15]. The reason for these differences between populations is unknown, although it is possible that they might depend on the prevalence in these populations of patients with autoantibody related forms of RA, in particular the prevalence of those who have pathogenic autoantibodies that directly interact with FcγRs (especially FcγRIIIa).

Anti-GPI antibodies are candidate arthritogenic antibodies. In K/B×N mice, polyclonal or two monoclonal anti-GPI antibodies induced arthritis in several strains of mice [16]. Moreover, FcγRIII deficient mice were resistant to anti-GPI antibody induced arthritis [3]. Another recent report [17] also confirmed that immune complex and FcγRIII are essential initiators of arthritis through sequential activation of effector cells, thus giving antibodies access into the joint. In human RA, anti-GPI antibodies have frequently been detected in patients with aggressive forms of arthritis [18,19], and their levels correlated significantly with extra-articular manifestations such as rheumatoid nodules, rheumatoid vasculitis and Felty's syndrome [20]. Moreover, a modest association of homozygosity for the *FCGR3A*-158V allele with RA in the nodular phenotype was suggested by Morgan and coworkers [11], suggesting the presence of a link between anti-GPI antibodies and *FCGR3A *allele. However, whether anti-GPI antibody positive status correlates with RA is a matter of controversy [18-22]. In our assay few healthy individuals retained anti-GPI antibodies; however, we do not know whether these protective phenotypes are associated with certain human gene polymorphisms.

In order to determine the relationship between functional polymorphisms of *FCGR *and possible arthritogenic anti-GPI antibodies in human conditions, we examined the correlation of these polymorphisms with anti-GPI positivity.

## Materials and methods

### Patients

The study was approved by the local ethics review committee and written informed consent was obtained from all participants. Blood samples were collected from 187 Japanese patients with RA (mean age 46 ± 17 years; 33 females; mean disease duration 12.9 years [range 1–46 years]) including four with vasculitis and three with Felty's syndrome. These patients, randomly selected from among patients visiting the clinic, were followed at University of Tsukuba Hospital. The diagnosis of RA was based on the criteria presented by the American College of Rheumatology [23]. In addition, 158 Japanese volunteers (mean age 30 ± 9 years; 105 females) were recruited from our institute to serve as a healthy comparison group. All healthy individuals were free of rheumatic disease symptoms, and derived from the same geographic locations.

### Enzyme-linked immunosorbent assay for GPI

In order to select anti-GPI antibody positive patients, we used recombinant human GPI (described in detail previously [18]) or rabbit muscle GPI (Sigma, St Louis, MO, USA). Both antigens were used at 5 μg/ml (diluted in phosphate-buffered saline [PBS]) to coat microtitre plates (12 hours, 4°C). After washing twice with washing buffer (0.05% Tween 20 in PBS), Block Ace (diluted 1/4 in 1 × PBS; Dainippon Pharmaceuticals, Osaka, Japan) was used for saturation (30 min at 37°C). After two washes, sera (diluted 1/50) were added and the plates were incubated for 12 hours at 4°C. After washing, alkaline phosphatase (AP)-conjugated anti-human IgG (Fc fragment specific; Jackson Immuno Research, West Grove, PA, USA) was added to the plate (dilution 1/1000, for 1 hour at room temperature). After three washes, colour was developed with AP reaction solution (containing 9.6% diethanol amine, 0.25 mmol/l MgCl_2_; pH 9.8) with AP substrate tablets (Sigma; one AP tablet per 5 ml AP reaction solution). Plates were incubated for 1 hour at room temperature, and the optical density (OD) was measured by plate spectrophotometry at 405 nm. Determinations were performed in triplicate and standardized between experiments by reference to a highly positive human anti-GPI serum. The primary reading was processed by subtracting OD readings of control wells (coated with gluthathione-S-transferase (GST) and Block Ace for recombinant GPI–GST and rabbit GPI, respectively). The cutoff OD was calculated from the ELISA reactions of 158 healthy Japanese donors. Those who were double positive to both antigens were considered anti-GPI antibody positive. Because we used two antigens for the discrimination, the cutoff OD (mean value + 1 standard deviation) was 0.98 for human recombinant GPI and 0.64 for rabbit native GPI.

**Figure 1:**
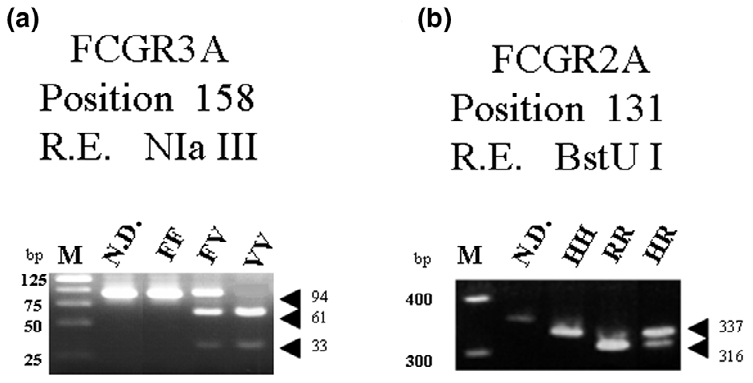
PCR-RFLP analysis of the *FCGR3A *and *FCGR2A *genes. cDNA was amplified with primers and restriction digested using appropriate enzymes. Digested PCR products were visualized with ethidium bromide. **(a) ***FCGR3A *gene and **(b) ***FCGR2A *gene. ND, nondigested PCR product; RE, restriction enzyme.

Genomic DNA was isolated from 0.5 ml anticoagulated peripheral blood, from 187 RA patients and 158 healthy individuals, by using DNA QuickII DNA purification kit (Dainippon Pharmaceuticals, Osaka, Japan). FcγR polymorphisms (*FCGR3A*-158V/F) were identified, as described by Koene and coworkers [6], using a nested PCR followed by allele specific restriction enzyme digestion. For homozygous FcγRIIIA-158F patients only one undigested band (94 bp) was visible. Three bands (94 bp, 61 bp and 33 bp) were seen in heterozygous individuals, whereas for homozygous FcγRIIIA-158V patients only two digested bands (61 bp and 33 bp) were detected (Fig. 1a). These genotyping findings were confirmed by direct sequencing in some individuals.

### FcγRIIA-131H/R genotyping

Genotyping of FcγRIIA-131H/R also consisted of PCR followed by an allele specific restriction enzyme digestion, in accordance with the method reported by Jiang and coworkers [24]. The *FCGR2A*-131H and *FCGR2A*-131R alleles were visualized as 337 bp and 316 bp DNA fragments, respectively (Fig. 1b). These genotyping findings were confirmed by direct sequencing in some individuals.

### Statistical analysis

The data were analyzed using the Student's *t*-test and the χ^2 ^test, and Fisher's exact test was used when expected frequencies were lower than 5. We used Mann–Whitney U-test to evaluate the distribution of anti-GPI antibodies in FcγRIIIA-158V/V RA patients and healthy individuals. *P *< 0.05 was considered statistically significant.

## Results

**Figure 2:**
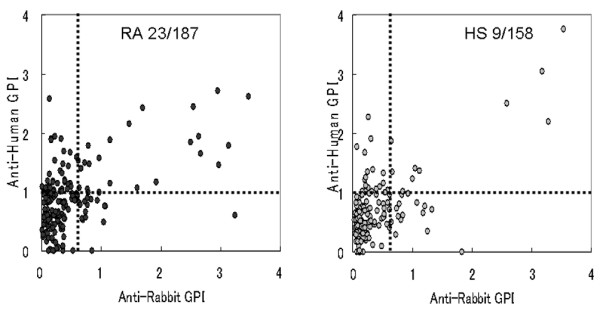
Population of anti-GPI antibody positive individuals, and *FCGR3A *and *FCGR2A *genotypes. The study included 187 patients with rheumatoid arthritis and 158 healthy Japanese individuals. The horizontal and vertical dotted lines represent the cutoff optical density values calculated from ELISA reactions of 158 healthy individuals for human recombinant GPI and rabbit native GPI, respectively. Individuals positive for both antibodies were considered anti-GPI antibody positive. Numbers in each graph represent the proportions of individuals positive for anti-GPI antibodies relative to the total number of individuals in that group. GPI, glucose-6-phosphate isomerase; HS, healthy subjects; RA, rheumatoid arthritis.

Our ELISA assay is highly specific because we used recombinant bacterial human GPI and native rabbit GPI, and double positivity for the two antibodies correlated significantly with the results of western blotting to GPI [18]. Because two GPI antigens were used for discrimination, the cutoff value of the OD was the mean value + one standard deviation from 158 healthy individuals, estimated using ELISA. Those who were positive for both antibodies were considered to be anti-GPI antibody positive. Using these definitions, 23 (12.3%) RA patients were anti-GPI antibody positive, and nine (5.7%) healthy individuals were anti-GPI antibody positive (Fig. 2). Statistical analysis revealed a significant difference in anti-GPI antibody positivity between RA patients and healthy individuals (χ^2 ^= 4.438, with one degree of freedom; *P *= 0.0352).

To analyze whether functional *FCGR *polymorphisms were correlated with anti-GPI antibody positive and negative individuals, we performed *FCGR *genotyping. *FCGR3A *and *FCGR2A *genotypes in the control group were in Hardy–Weinberg equilibrium. The *FCGR3A*-158V allele (high affinity genotype) was more frequently identified in patients with RA than in healthy individuals within the anti-GPI antibody positive population (χ^2 ^= 0.012, with one degree of freedom; *P *= 0.012; Tables 1 and 2). In addition, these differences were evident when individuals were categorized according to the presence or absence of these genotypes: 56.5% of patients with RA were homozygous or heterozygous with respect to *FCGR3A*-158V, as compared with 11.1% of healthy individuals; and 43.5% of patients with RA were homozygous with respect to *FCGR3A*-158F, as compared with 88.9% of healthy individuals (χ^2 ^= 5.42 with one degree of freedom; *P *< 0.02; Tables 1 and 2). Comparison of *FCGR3A*-158V allele frequency between RA patients and healthy individuals revealed no statistically significant difference: 48.7% of patients with RA were homozygous or heterozygous with respect to *FCGR3A*-158V, as compared with 42.4% of healthy individuals; and 51.3% of patients with RA were homozygous with respect to *FCGR3A*-158F, as compared with 57.6% of healthy individuals (χ^2 ^= 1.04 with one degree of freedom; *P *= 0.245; Table 1).

Next, *FCGR2A *genotyping was conducted in the same cohort (Table 1). In contrast to *FCGR3A*, the frequency of the *FCGR2A*-131H allele (high affinity genotype) was not significantly different between the two groups within the anti-GPI antibody positive population (χ^2 ^= 0.862 with one degree of freedom; *P *= 0.35; Tables 1[Table T1] and 2). These differences were also not evident when individuals were categorized according to the presence or absence of these genotypes (*P *= 0.19; Tables 1 and 3).

We also analyzed the association between FcγR and other related autoantibodies such as RF. There was no difference between RF positive and RF negative populations of RA patients (*P *= 0.82 and *P *= 0.4 for *FCGR3A *and *FCGR2A*, respectively; Table 4).

**Figure 3:**
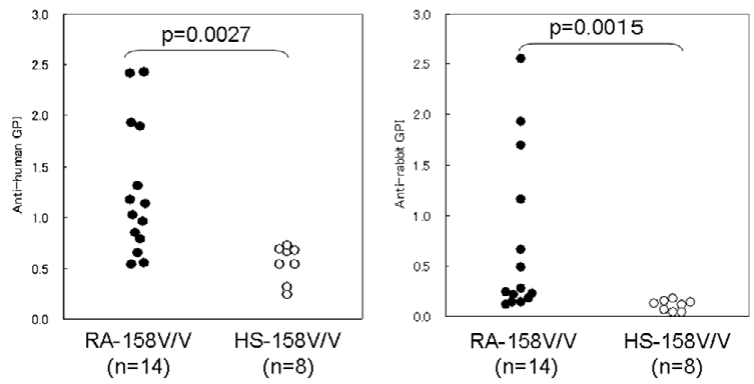
Higher titres of anti-human and anti-rabbit GPI antibodies in *FCGR3A*-158V/V RA patients versus healthy individuals. In individuals homozygous for the *FCGR3A *high affinity V/V genotype (14 RA patients and 8 healthy individuals), both anti-human GPI antibodies and anti-rabbit GPI antibodies were significantly elevated in the RA group (*P *= 0.0027 and *P *= 0.0015 for anti-human GPI antibodies and anti-rabbit GPI antibodies, respectively, by Mann–Whitney U-test). GPI, glucose-6-phosphate isomerase; RA, rheumatoid arthritis.

Finally, in order to identify the relationship between *FCGR3A*-158V allele and anti-GPI antibodies more clearly, we focused on individuals who were homozygous for the high affinity *FCGR3A*-158V/V genotype (14 RA patients and eight healthy individuals) and compared their anti-GPI antibody titres. Surprisingly, both anti-human GPI antibodies and anti-rabbit GPI antibodies were significantly elevated in the RA group (*P *= 0.0027 and *P *= 0.0015 for anti-human GPI antibodies and anti-rabbit GPI antibodies, respectively, by Mann–Whitney U-test; Fig. 3). This suggests that anti-GPI antibody positivity might predispose individuals with the *FCGR3A*-158V/V genotype to arthritis.

## Discussion

Several studies have indicated that anti-GPI antibodies are potential arthritogenic antibodies [18-20] because they were frequently detected in patients with severe forms of RA. Because high titres of these antibodies (IgG, not IgM) were also detected in healthy individuals, the arthritogenicity of these antibodies should be due to modulation – by the low affinity genotype of FcγRs – of the bypass between immune complex and FcγR bearing cells. In a GPI immunized mouse model severe arthritis occurred only in DBA/1 mice, although the production of anti-GPI antibodies was almost equal in arthritis susceptible and resistant mouse strains [25]. Thus, the incidence of arthritis might depend on certain genetic factors such as FcγR. Anti-GPI antibody positive individuals express several GPI variant mRNAs in peripheral blood monocytes [26]. This observation supports the notion that the presence of GPI variants is necessary to produce anti-GPI autoantibodies, and that genetic factors such as FcγRIIIA are important in the development of arthritis. Based on this conclusion, it is conceivable that the production of anti-GPI antibodies does not occur as a 'result' of joint destruction.

Our results do not indicate that individual polymorphisms in the *FCGR3A *and *FCGR2A *genes play roles in susceptibility to RA. Despite the lack of association with individual *FCGR *polymorphisms in the whole cohort, our studies suggest that *FCGR3A*-158V/F polymorphisms play a crucial role in RA among those individuals who are positive for anti-GPI antibodies (Tables 2 and 3). Moreover, focusing on *FCGR3A*-158V/V homozygous individuals, anti-GPI antibodies were clearly evident in patients with RA. These findings suggest that anti-GPI antibodies might have arthritogenic potential in individuals homozygous for *FCGR3A*-158V/V.

## Conclusion

Our findings show that *FCGR3A*-158V/F functional polymorphisms were associated with RA among anti-GPI antibody positive individuals. This is the first report on possible mechanisms of arthritic diseases; they are tightly regulated by some genes, especially by FcγR genotype, as well as by production of arthritogenic autoantibodies.

## Abbreviations

AP = alkaline phosphatase; bp = base pairs; ELISA = enzyme-linked immunosorbent assay; FcγR = Fcγ receptor; GPI = glucose-6-phosphate isomerase; GST = gluthathione-S-transferase; OD = optical density; PBS = phosphate-buffered saline; PCR = polymerase chain reaction; RA = rheumatoid arthritis; RF = rheumatoid factor.

## Competing interests

The author(s) declare that they have no competing interests.

## Authors' contributions

IM wrote the manuscript and conceived the study. HZ performed FcγR genotyping and coordinated the statistical analysis. YM, TY and YK performed GPI ELISA. TH participated in clinical assessment. TS participated in the full design and coordination of the study, and DG, SI and AT participated in writing the discussion.

## Figures and Tables

**Table 1 T1:** Frequencies of *FCGR3A *and *FCGR2A *genotypes in patients with RA and positive and negative for anti-GPI antibodies

	*FCGR3A*-158			*FCGR2A*-131		
	
	FF low	F/V	VV high	HH high	H/R	RR low
GPI^+ ^RA (*n *= 23)	10 (43.5)	9 (39.1)	4 (17.4)	16 (69.6)	6 (26.1)	1 (4.3)
GPI^- ^RA (*n *= 164)	86 (52.4)	68 (41.5)	10 (6.1)	128 (78)	29 (17.7)	7 (4.3)
GPI^+ ^Control (*n *= 9)	8(88.9)	1 (11.1)	0 (0)	4 (44.4)	5 (55.6)	0 (0)
GPI^- ^Control (*n *= 149)	83 (55.7)	58 (38.9)	8 (5.4)	109 (73.2)	40 (26.8)	0 (0)

Data are expressed as number (percentage) of individuals. GPI, glucose-6-phosphate isomerase; high, high affinity genotype; low, low affinity genotype; RA, rheumatoid arthritis.

**Table 2:** Alleic skewing of *FCGR3A *and *FCGR2A *in anti-GPI antibody positive healthy individuals

Polymorphism	Allele	RA GPI^+ ^(*n *= 46)	Healthy GPI^+ ^(*n *= 18)	*P *(χ^2^)	*P *(Fisher's)	OR (95% CI)
*FCGR3A*-158	F	29	17	0.012	0.013	0.10 (0.01–0.82)
	V	17	1			
*FCGR2A*-131	H	38	13	0.35	0.4902	1.83 (0.51–6.59)
	R	8	5			

*P *values are given for RA versus healthy individuals using a 2×2 contingency table. CI, confidence interval; Fisher's, Fisher's probability test; OR, odds ratio; RA, rheumatoid arthritis.

**Table 3:** Genotype skewing of *FCGR3A *and *FCGR2A *gene polymorphisms in anti-GPI antibody positive healthy individuals

Polymorphism	Genotype	RA GPI^+ ^(*n *= 23)	Healthy GPI^+ ^(*n *= 9)	*P *(χ^2^)	*P *(Fisher's)	OR (95% CI)
*FCGR3A*-158	FF	10 (43.5%)	8 (88.9%)	0.019	0.044	0.09 (0.01–0.89)
	FV/VV	13(56.5%)	1 (11.1%)			
*FCGR2A*-131	HH	16 (69.6%)	4(44.4%)	0.19	0.24	2.86 (0.58–13.96)
	HR/RR	7 (30.4%)	5 (55.6%)			

*P *values are given for RA versus healthy individuals using a 2×2 contingency table. CI, confidence interval; Fisher's, Fisher's probability test; OR, odds ratio; RA, rheumatoid arthritis.

**Table 4:** *FCGR3A *and *FCGR2A *genotypes in rheumatoid factor positive and negative RA patients

Polymorphism	Genotype	RA RF^+ ^(n = 130)	RA RF^- ^(*n *= 57)	*P *(χ^2^)	OR (95% CI)
*FCGR3A*-158	FF	66 (50.8%)	30(52.6%)	0.82	0.93 (0.50–1.73)
	FV/VV	64(49.2%)	27 (47.4%)		
*FCGR2A*-131	HH	103 (79.2%)	42(73.7%)	0.4	1.36 (0.66–2.82)
	HR/RR	27 (20.8%)	15 (26.3%)		

*P *values are given for RA RF^+ ^versus RA RF^- ^using a 2×2 contingency table. CI, 95% confidence interval; OR, odds ratio; RA, rheumatoid arthritis; RF, rheumatoid factor.
